# Spatiotemporal association between COVID-19 incidence and type 1 diabetes incidence among children and adolescents: a register-based ecological study in Germany

**DOI:** 10.3389/fendo.2023.1287354

**Published:** 2024-01-03

**Authors:** Joachim Rosenbauer, Anna Stahl-Pehe, Christina Baechle, Stefanie Lanzinger, Clemens Kamrath, Oliver Kuß, Reinhard W. Holl

**Affiliations:** ^1^Institute for Biometrics and Epidemiology, German Diabetes Center, Leibniz Center for Diabetes Research at Heinrich Heine University Düsseldorf, Düsseldorf, Germany; ^2^German Center for Diabetes Research (DZD), Munich, Germany; ^3^Institute of Epidemiology and Medical Biometry, Central Institute for Biomedical Technology (ZIBMT), Ulm University, Ulm, Germany; ^4^Center of Child and Adolescent Medicine, Justus Liebig University Giessen, Giessen, Germany; ^5^Centre for Health and Society, Medical Faculty and University Hospital, Heinrich Heine University Düsseldorf, Düsseldorf, Germany

**Keywords:** coronavirus disease 2019 (COVID-19), type 1 diabetes, incidence rate, children and adolescents, epidemiology, registry

## Abstract

**Objective:**

Studies have shown an increased incidence of pediatric type 1 diabetes during the COVID-19 pandemic, but the detailed role of SARS-CoV-2 infection in the incidence increase in type 1 diabetes remains unclear. We investigated the spatiotemporal association of pediatric type 1 diabetes and COVID-19 incidence at the district level in Germany.

**Methods:**

For the period from March 2020 to June 2022, nationwide data on incident type 1 diabetes among children and adolescents aged <20 years and daily documented COVID-19 infections in the total population were obtained from the German Diabetes Prospective Follow-up Registry and the Robert Koch Institute, respectively. Data were aggregated at district level and seven time periods related to COVID-19 pandemic waves. Spatiotemporal associations between indirectly standardized incidence rates of type 1 diabetes and COVID-19 were analyzed by Spearman correlation and Bayesian spatiotemporal conditional autoregressive Poisson models.

**Results:**

Standardized incidence ratios of type 1 diabetes and COVID-19 in the pandemic period were not significantly correlated across districts and time periods. A doubling of the COVID-19 incidence rate was not associated with a significant increase in the incidence rate of type 1 diabetes (relative risk 1.006, 95% CI 0.987; 1.019).

**Conclusion:**

Our findings based on data from the pandemic period indirectly indicate that a causal relationship between SARS-COV-2 infection and type 1 diabetes among children and adolescents is unlikely.

## Introduction

During the COVID-19 pandemic, an increase in the incidence of pediatric type 1 diabetes was observed (1–5). Despite several hypotheses being discussed (e.g., direct ß-cell destruction, accelerated ß-cell autoimmunity, hyperinflammation, infection of pancreatic microvasculature, secondary effects of pandemic containment measures), the detailed role of SARS-CoV-2 infection in the increase in type 1 diabetes remains unclear (4–7). If there is a causal association between SARS-CoV-2 infection and new-onset type 1 diabetes, spatiotemporal relationships between the occurrence of both diseases should be detectable. Therefore, the aim of this study was to assess the spatiotemporal association of COVID-19 incidence with type 1 diabetes incidence among children and adolescents in Germany during the COVID-19 pandemic.

## Materials and methods

We followed the STROBE reporting guidelines for this ecological, register-based observational study.

### Data sources, spatiotemporal aggregation of data

For the period from March 2020 to June 2022, nationwide data on incident type 1 diabetes among children and adolescents aged younger than 20 years were selected from the German Diabetes Prospective Follow-up Registry (DPV) (8). Locally collected longitudinal data are transmitted in pseudonymized form to the University of Ulm, Ulm, Germany, for central plausibility checks and analyses. Inconsistent data are reported back to the participating centers for validation and/or correction. The data are then completely anonymized for analysis (8). The long-term coverage of the DPV for pediatric patients with type 1 diabetes has recently been estimated at 93% (9). The ethics committee of the University of Ulm approved the analysis of anonymized DPV data (no. 314/21). Participating DPV centers are responsible for obtaining informed consent from patients or their guardians (2, 3, 8, 9). Diabetes type was initially determined by the treating physicians at the diagnosis of diabetes based on clinical presentation, patient characteristics and laboratory results like diabetes-related autoantibody measurements, and, if necessary, revised at the biannual DPV data update based on additional clinical and laboratory data in the course of the disease, according to national guidelines [German Diabetes Association (10), which are consistent with International Society for Pediatric and Adolescent Diabetes (ISPAD) diagnostic criteria (11)]. By November 15, 2022, 267 centers in Germany had registered at least one patient with type 1 diabetes in the DPV for the observation period. Incident cases were assigned to 400 districts based on the residential post codes at diagnosis.

For the same period, nationwide data on daily documented COVID-19 infections in the total population at the district level were obtained from the Robert Koch Institute (RKI) (12). Verification of COVID-19 infections was based on positive results of specific PCR tests.

For each district, type 1 diabetes and COVID-19 data were aggregated across seven time periods that were defined according to COVID-19 pandemic waves (March 2020 – April 2020, May 2020 – August 2020, September 2020 – May 2021, June 2021 – July 2021, August 2021 – December 2021, January 2022 – April 2022, and May 2022 – June 2022).

### Covariates

Age- and sex-specific population data, geographical data for mapping, an indicator of area deprivation [German Index of Socioeconomic Deprivation (GISD) (13)], and an indicator of urban/rural typology, each at district level, were obtained from Federal Offices (14, 15), the RKI (16) and EUROSTAT (17), respectively.

The area deprivation index GISD was categorized into quintiles (Q), with Q1 and Q5 corresponding to the least and most deprived districts, respectively. The urban/rural typology distinguished predominantly urban, intermediate, and predominantly rural districts. A schematic overview defining the urban/rural typology at the district level (nomenclature of territorial units for statistics (NUTS) level 3 regions) is given at EUROSTAT (18).

### Data analysis

District-level type 1 diabetes incidence rates were indirectly age- and sex-standardized using age- (0–4, 5–9, 10–14, 15–19 years) and sex-specific national type 1 diabetes incidence rates over the full observation period as the reference incidence rates to calculate standardized incidence ratios (T1D-SIRs). Because age- and sex-specific numbers of COVID-19 cases were not available, district-level COVID-19 incidence rates were indirectly standardized using the nationwide COVID-19 incidence over the total observation period as the reference incidence to calculate standardized incidence ratios (COVID-19-SIRs).

Spearman correlation analysis with 95% confidence intervals (CIs) based on Fisher’s z-transformation and six Bayesian spatiotemporal conditional autoregressive (CAR) Poisson models (19), each accounting for a different spatiotemporal autocorrelation structure, were applied to assess the association between the COVID-19-SIR and T1D-SIR adjusted for area deprivation and urban/rural typology. Details on the Bayesian spatiotemporal CAR Poisson models and the estimation method are given in the **Supplementary Material**. The results are presented as posterior means and 95% credible intervals. All analyses were performed using SAS Version 9.4 (SAS Institute Inc., Cary, North Carolina) and R Version 4.2.2, including the CARBayesST R package (19, 20).

## Results

Between March 2020 and June 2022, 9,596 children and adolescents (mean (SD) age at onset 9.4 (4.3) years, 5347 (55.7%) boys, mean BMI 19.8 (3.9) kg/m^2^) with newly diagnosed type 1 diabetes were identified. The overall incidence of type 1 diabetes was 26.8 (95% confidence interval 26.3; 27.4) per 100,000 person-years. A total of 28.4 million individuals with COVID-19 were identified. The overall incidence of COVID-19 was 146.19 (146.13; 146.24) per 1,000 person-years.

**Figure 1:**
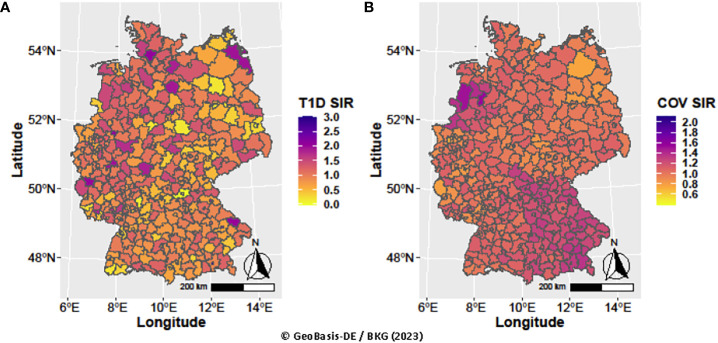
Spatial distribution of type 1 diabetes and COVID-19 standardized incidence ratios averaged over periods **(A)** T1D SIR and **(B)** COV SIR, respectively.

**Figure 2:**
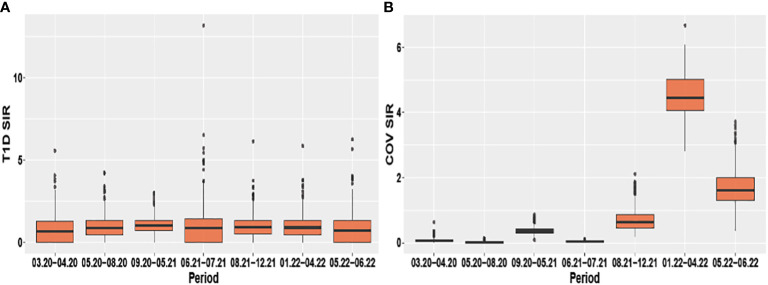
Boxplots of the spatio-temporal distribution of type 1 diabetes and COVID-19 standardized incidence ratios **(A)** T1D SIR and **(B)** COV SIR respectively stratified by period.

Evaluating pandemic data from March 2020 to June 2022, neither the period-averaged spatial (**Figure 1**) nor the spatiotemporal patterns of the T1D-SIR revealed an apparent association with the patterns **Figure 2** of the COVID-19-SIR. Concordantly, T1D-SIRs and COVID-19-SIRs were not significantly correlated across districts (period-averaged SIRs, n=400, r=0.032 (-0.066; 0.130) or across districts and time periods (n=2800, r=0.009 [-0.028; 0.046]). Furthermore, the best fitting spatiotemporal CAR Poisson model (CARanova) showed that a doubling of the COVID-19-SIR was not associated with a significant increase in T1D-SIR (relative risk 1.006 (95% credible interval 0.987; 1.019). The alternative models resulted in similar relative risk estimates (**Table 1**).

**Table 1:** Model selection results and relative risk estimates of type 1 diabetes from spatio-temporal models for type 1 diabetes cases dependent on the COVID-19 standardized incidence ratio.

CAR Poisson Model^a^	DIC	p.d	WAIC	p.w	Relative risk (95% CI)^b^
CARlinear	9893.5	170.3	9901.3	164.6	1.007 (0.995; 1.019)
CARanova^c^	**9874.5**	**210.1**	**9878.3**	**195.3**	**1.006 (0.987; 1.027)**
CARsepspatial	10046.1	170.6	10054.3	167.2	1.002 (0.980; 1.023)
CARar	9899.3	234.6	9904.6	216.4	1.005 (0.994; 1.018)
CARadaptive	9891.0	217.2	9894.3	200.9	1.004 (0.986; 1.018)
CARlocalised	9889.3	200.5	9909.8	204.1	1.022 (0.990; 1.053)

CAR Model, conditional autoregressive model; DIC, Deviance Information Criterion; WAIC, Watanabe-Akaike Information Criterion; p.d and p.w, estimated effective number of parameters corresponding DIC and WAIC, respectively; 95% CI, 95% credible interval; SIR, standardized incidence ratio; best fitting model indicated in bold face.

a dependent variable: log(type 1 diabetes cases); offset: log(expected type 1 diabetes cases); independent variables: log(COVID-19-SIR), area deprivation quintiles, EUROSTAT urban/rural typology ; spatio-temporal auto(-correlated) random effects which are specifically modelled according to the CAR Poisson model applied.

b Increase in T1D-SIR per doubling of COVID-19-SIR according to posterior mean and posterior 95% credible interval (corresponding to the posterior 2.5% and 97.5% quantiles).

c best fitting model according to DIC and WAIC.

## Discussion

Our findings based on pandemic data from March 2020 to June 2022 suggest that COVID-19 incidence was not associated with type 1 diabetes incidence among children and adolescents, which is consistent with results from recent observational cohort studies (21–23). A nationwide German study found no evidence that the observed increase in type 1 diabetes incidence during the COVID-19 pandemic was associated to direct effects of the COVID-19 pandemic (21). A nationwide Finnish study observed that only 0.9% (5 of 583) of patients with type 1 diabetes diagnosed during the pandemic tested positive for infection-induced SARS-CoV2 antibodies, and concluded that the observed increase in type 1 diabetes incidence during the pandemic could be a consequence of lockdown measures rather than a direct effect of SARS-CoV-2 infection (22). Consistently, a nationwide prospective study in Denmark, in which 90% of children were tested for SARS-CoV-2 infection, found no increased risk of type 1 diabetes after SARS-CoV-2 infection [hazard ratio 0.85 (95% CI 0.80–1.04)] (23). Further, three studies found no evidence for a role of SARS-CoV2 infection in the development of type 1 diabetes autoimmunity (24–26). The study by Rewers et al. estimated a non-significant odds ratio of 1.06 (95% CI: 0.59; 1.80) for the presence of multiple islet autoantibodies after a SARS-CoV-2 infection (24). However, it should be noted that some studies reported an increased risk of type 1 diabetes subsequent to a SARS-CoV-2 infection (27, 28). One meta-analyses including three studies reported a 42% increased risk for type 1 diabetes [RR 1.42 (95% CI 1.38; 1.46)] after a SARS-CoV-2 infection (27), a second meta-analysis comprising 10 studies estimated a 62% increased risk [RR 1.622 (95% CI 1.347; 1.953)] (28). However, many studies suggesting a direct effect of SARS-CoV-2 infection on the type 1 diabetes risk had methodological weaknesses, in particular because asymptomatic SARS-CoV-2 infections were not adequately considered (28). A most recent study reported a significant temporal association of SARS-CoV-2 infection with the development of islet autoimmunity in young children with high genetic risk for Type 1 diabetes [Hazard ratio 3.5 (95% CI: 1.6; 7.7)] (29). Thus, evidence for a direct SARS-CoV-2-effect for the development of islet autoimmunity and type 1 diabetes remains inconsistent.

The strengths of this study include the national registration of type 1 diabetes and COVID-19 incidence rates and application of various CAR Poisson models that accounted for spatiotemporal dependencies. However, some residual bias may remain due to unmeasured covariates; so we did not consider changes in BMI, physical activity or diet during the pandemic in the present study. However, changes in BMI-Z-scores and physical activity in children, adolescents and young adults with type 1 diabetes documented in the DPV Registry have been investigated in two recent studies. The findings showed a reduction in physical activity but no acceleration of BMI-Z-scores among young people (6-21 years) (30), or at most a slight acceleration of BMI-Z-scores in prepubertal children only (31). We believe that adjusting for these possible confounders is unlikely to affect our results seriously. Further, we did not analyze diabetes-related autoantibodies in this study. However, the rate of diabetes-related autoantibody positivity has been evaluated during the pandemic period several times. According to the most recent analysis based on the DPV registry data from 2020-2021, about 73-74% of children with incident type 1 diabetes in Germany are screened for diabetes-related autoantibodies, and 92-93% of the screened children are positive for at least one diabetes-related antibody (3). Of note, an effect of the COVID-19 pandemic on the autoantibody positive rate or the frequency of autoantibody negative type 1 diabetes was not observed (2, 3, 28, 32).

Further, we could not consider individual SARS-Cov-2 infection and respective corticosteroid therapy in children and adolescents with newly diagnosed type 1 diabetes because these data were not regularly documented in the DPV registry. However, SARS-Cov-2 infection is mostly not severe and often asymptomatic in children and adolescents. Therefore, the proportion of children receiving corticoid therapy can be assumed to be small and thus responsible for incident diabetes in few cases at most. This will not severely have biased our findings.

Lockdown measures and home schooling certainly affected the frequency of SARS-COV-2 infection in the population and possibly the diabetes risk [e.g. through changes in exposure to common viral infections, stress levels and possibly various other factors (2)]. In Germany, periods of lockdown measures with home schooling were from 22 March 2020 until beginning of May 2020 and from the mid of December 2020 until 10th of May 2021 (partial home schooling). But periods and measures varied regionally across German federal states or even districts depending on population COVID-19 incidence levels. However, all these conditions are unlikely to affect our results seriously because we analyzed spatiotemporal associations between COVID-19 and type 1 diabetes incidence rates at the district level over several pandemic periods. Unfortunately, we could not evaluate spatiotemporal associations between type 1 diabetes and COVID-19 incidences for different age-groups, because age-specific COVID-19 data were not completely available at district level. An additional limitation is that we used a coarse temporal resolution and did not account for time lags between COVID-19 incidence and type 1 diabetes incidence; this fact may have masked possible associations.

In conclusion, our findings based on data from the pandemic period show no association between COVID-19 incidence in the total population and type 1 diabetes incidence among children and adolescents. Our study therefore indirectly indicates that a causal relationship between SARS-COV-2 infection and type 1 diabetes among children and adolescents is unlikely. Possible secondary pandemic effects should be investigated in further studies.

## Data availability statement

The raw data supporting the conclusions of this article will be made available by the authors, without undue reservation.

## Ethics statement

The ethics committee of the University of Ulm approved the analysis of anonymized data from the national DPV registry (no. 314/21).

## Author contributions

JR: Conceptualization, Formal Analysis, Funding acquisition, Investigation, Methodology, Project administration, Visualization, Writing – original draft, Writing – review & editing. AS: Writing – original draft, Writing – review & editing. CB: Writing – review & editing. SL: Writing – review & editing. CK: Supervision, Writing – review & editing. OK: Methodology, Supervision, Writing – review & editing. RH: Funding acquisition, Investigation, Supervision, Writing – review & editing.
