# Supplementary material for: Spatiotemporal association between COVID-19 incidence and type 1 diabetes incidence among children and adolescents: a register-based ecological study in Germany
Source: Front Endocrinol (Lausanne). 2024 Jan 3;14:1287354. doi: 10.3389/fendo.2023.1287354 (PMC10791930; doi:10.3389/fendo.2023.1287354)
Supplement: Supplementary file 1 [file DataSheet_1.docx]

**Supplementary Material**

Rosenbauer J, Stahl-Pehe A, Baechle C, Lanzinger S, Kamrath C, Kuß O, Holl RW (2023). Spatiotemporal association between COVID-19 incidence and type 1 diabetes incidence among children and adolescents: A register-based ecological study in Germany. Front. Endocrinol. 14:1287354. doi: 10.3389/fendo.2023.1287354

**Bayesian spatiotemporal conditional autoregressive (CAR) Poisson models**

The present analysis dataset included data from K=400 districts and T=7 time periods. The number of incident type 1 diabetes cases in spatial unit (district) k, 1 ≤ k ≤ K, in time period t, 1 ≤ t ≤ T, is assumed to be Poisson-distributed with expectation $\left( E_{kt}\times\lambda_{kt} \right)$, i.e. $Y_{kt}\sim Poisson\left( E_{kt}\times\lambda_{kt} \right)$, with $\lambda_{kt}$ representing the type 1 diabetes incidence for the spatial unit k and time period t, and $E_{kt}$ representing the age- and sex-standardized number of expected type diabetes cases for the spatial unit k and time period t assuming age- and sex-specific type 1 diabetes incidences equal to age- and sex-specific national type 1 diabetes incidences over the total observation period. The logarithm of the incidence $\lambda_{kt}$ was modeled as

$\log\left( \lambda_{kt} \right)=\mathbf{X}_{\mathbf{kt}}^{\boldsymbol{'}}\boldsymbol{\times}\boldsymbol{\beta}+\beta_{1}\times log\left( \text{COVID-19-SIR}_{kt} \right)\mathbf{+}\psi_{\mathrm{kt}},1 \leq k \leq K,1\leq t\leq T$,

with the matrix $X_{kt}$representing the confounders (area deprivation quintiles and urban/rural typology) and $\psi_{\mathrm{kt}}$ representing spatio-temporal auto-correlated random effects. Six different spatio-temporal auto-correlation structures were modelled:

$\psi_{kt}=\left\{ \begin{aligned} \phi_{k}+\left( \alpha+\delta_{k} \right)\times\frac{\left( t-\bar{t} \right)}{T}, CARlinear model \\ \phi_{k}+\delta_{t}+\gamma_{kt}, CARanova model \\ \phi_{kt}+\delta_{t}, CARsepspatial model, \\ \phi_{kt}, CARar model \\ \phi_{kt}, CARadaptive model \\ \phi_{kt}+\lambda_{Z_{kt}}, CARlocalised model \end{aligned} \right.1 \leq k \leq K,1\leq t\leq T$,

Detailed descriptions of the spatio-temporal random effects ($\phi_{k},\phi_{kt}, \delta_{k},\delta_{t},\gamma_{kt,}\lambda_{Z_{kt}}$) of the specific CAR models are given in Lee et al.^1^ Spatial autocorrelation is controlled by a non-negative K x K neighborhood/adjacency matrix that represents the spatial neighborhood structure of the spatial units. The CAR prior distribution was modelled according to Leroux^2^.

Regression parameters were estimated by Markov chain Monte-Carlo (MCMC) methods. Default conditional autoregressive spatio-temporal priors were assigned to the fixed effects (ß) and random effects.^3^ Each of the models was run with a burn-in of 20,000 iterations and further 100,000 iterations. After a thinning of 100 iterations to reduce auto-correlation in the MCMC samples the remaining 1,000 samples were used for inference. The best fitting model was selected based on the deviance information criterion (DIC) and the Watanabe-Akaike information criterion (WAIC).^4,5^ Results are presented as posterior means and posterior 95% credible intervals, corresponding to the 2.5% quantile and 97.5% quantile.

The Bayesian spatiotemporal CAR Poisson models were fitted using the CARBayesST R-package with R Version 4.2.2.^1,3,6^

**References**

1. Lee D, Rushworth A, Napier G, Pettersson W. *CARBayesST version 3.3.1: Spatio-Temporal Areal Unit Modelling in R with Conditional Autoregressive Priors.* 2023.

2. Leroux BG, Lei, X., Breslow, N. *Estimation of disease rates in small areas: A new mixed model for spatial dependence. In: Halloran, M.E., Berry, D. (Eds.), Statistical Models in Epidemiology, the Environment, and Clinical Trials.* Spinger-Verlag, New York, 2000, pp. 179-191.

3. Lee D, Rushworth A, Napier G, Pettersson W. *Package ‘CARBayesST.* 2023.

4. Spiegelhalter DJ, Best NG, Carlin BP, Van Der Linde A. Bayesian Measures of Model Complexity and Fit. *Journal of the Royal Statistical Society Series B: Statistical Methodology.* 2002;64(4):583-639.

5. Watanabe S. Asymptotic equivalence of Bayes cross validation and widely applicable information criterion in singular learning theory. *J Mach Learn Res.* 2010;11(116):3571–3594.

6. The R Project for Statistical Computing. R Foundation for Statistical Computing,; 2023. https://www.R-project.org/. Accessed 24.4.2023.
